# Enterovirus A71 Infection and Neurologic Disease, Madrid, Spain, 2016

**DOI:** 10.3201/eid2607.190037

**Published:** 2020-07

**Authors:** Pascal Del Giudice

**Affiliations:** Centre Hospitalier Intercommunal, Fréjus, France

**Keywords:** enterovirus 71, hand foot and mouth syndrome, purpura, viruses, enteroviruses, Spain, rashes, skin lesions, neurologic diseases

**To the Editor:** Taravilla et al. (*1*) observed mucocutaneous manifestations of 6 children with enterovirus, and “the main manifestation was petechial rash on the extremities.” This finding is unusual for enterovirus A71 (EV-A71) infections. Cutaneous manifestations of EV-A71 are reported as vesicular skin lesions, not purpura (*2*). It is important that physicians distinguish between petechial and vesicular rashes because petechial rashes in children are linked to other infectious agents, including parvovirus B19 and (especially within the context of encephalitis) *Neisseria meningitidis*. A precise dermatologic description is necessary for a correct clinical diagnosis. A too-rapid clinical examination might result in confusion between vesicular rash and petechial rash, especially when vesicles are very small. To avoid confusion, the authors should have provided high-quality clinical photos of the rashes, skin biopsy results, or both before considering EV-A71 as a new cause of febrile petechial rash in children.
